# Sequence assignment for low-resolution modelling of protein crystal structures

**DOI:** 10.1107/S2059798319009392

**Published:** 2019-07-31

**Authors:** Grzegorz Chojnowski, Joana Pereira, Victor S. Lamzin

**Affiliations:** a European Molecular Biology Laboratory, c/o DESY, Notkestrasse 85, 22607 Hamburg, Germany

**Keywords:** model building, sequence assignment, *ARP*/*wARP*, macromolecular crystallography, loop building, low-resolution modelling

## Abstract

The performance of automated model building in crystal structure determination usually decreases with the resolution of the experimental data, and may result in fragmented models and incorrect side-chain assignment. Presented here are new methods for machine-learning-based docking of main-chain fragments to the sequence and for their sequence-independent connection using a dedicated library of protein fragments. The combined use of these new methods noticeably increases sequence coverage and reduces fragmentation of the protein models automatically built with *ARP*/*wARP*.

## Abstract 2

Recent advances in automated protein model building using *ARP*/*wARP* are presented. The new methods include machine-learning-enhanced sequence assignment and loop building using a fragment database.

## 1. Introduction   

Model building is a key step in crystallographic structure determination. When the resolution of the X-ray diffraction data is better than 3.0 Å and the initial map is of reasonable quality, model building can often be accomplished straightforwardly using automated approaches. Automation may not just considerably accelerate the process of obtaining a macromolecular model, but may also make it more robust and reliable, thus helping to minimize human-dependent subjective interpretation (Weiss *et al.*, 2016 ▸). In difficult cases automated approaches may not fully succeed, but may still help to improve the electron-density maps to a level that enables unambiguous manual interpretation. This is particularly important owing to recent advancements in molecular-replacement and experimental phasing pipelines [examples include *BALBES* (Long *et al.*, 2008 ▸), *MrBUMP* (Keegan & Winn, 2007 ▸), *MORDA* (Vagin & Lebedev, 2015 ▸), *ARCIMBOLDO* (Sammito *et al.*, 2014 ▸) and *Auto-Rickshaw* (Panjikar *et al.*, 2009 ▸)], where automated model building is often used for the evaluation of plausible solutions (Ha & Boggon, 2018 ▸). However, in the presence of significant phase error and/or with limited resolution of the experimental data (worse than 2.5 Å) the model-building task remains challenging and, with the need for manual intervention, becomes time-consuming even for an experienced crystallographer.

The performance of automated model-building methods in crystallography decreases at lower resolution owing to the reduced information content that is present in the experimental data. The built backbone models become fragmented, which in turn complicates their docking to the target sequence as well as the completion of poorly resolved loop regions. The interpretation of lower resolution electron-density maps is usually addressed by the use of larger search objects. These may include secondary-structural elements for the initial interpretation of the maps with FFT-based template matching in reciprocal space (Terwilliger, 2003*a* ▸; Sheldrick, 2010 ▸), real-space pattern recognition (Langer *et al.*, 2008 ▸) or graph-based approaches (Chojnowski *et al.*, 2015 ▸). Jones & Thirup (1986 ▸) demonstrated that given the approximate C^α^ coordinates of a protein, all full main-chain atoms can be reconstructed using short fragments derived from a database of other proteins. Fragment libraries have also been proposed for the extension of protein chains (Terwilliger, 2003*a* ▸) and model completion (Cowtan, 2012 ▸).

The chain fragments modelled during main-chain tracing are subsequently matched to the sequence. This step not only helps to increase the completeness of the model but, more importantly, can identify gaps between the chain fragments for subsequent completion using sequence information. The residue types are usually identified by the analysis of a sparse-density representation and an exhaustive side-chain rotamer search (Langer *et al.*, 2008 ▸) and electron-density templates (Terwilliger, 2003*b* ▸; Cowtan, 2008 ▸). These approaches provide excellent results at high and medium resolution when the experimental X-ray data provide a sufficiently high observation-to-parameter ratio. However, their performance is considerably reduced at lower resolution (usually worse than 2.5 Å; Porebski *et al.*, 2016 ▸).

Automated model building using *ARP*/*wARP* proceeds in an iterative manner when main-chain fragments are identified and built in a density map, followed by their docking to the known protein sequence (Langer *et al.*, 2008 ▸). The sequence-assignment and side-chain building method originally implemented in *ARP*/*wARP* (*snow*) is based on the topology of the sparse-density representation using free atoms and an exhaustive side-chain conformational search around each C^α^ atom in a built main-chain fragment (Cohen *et al.*, 2004 ▸). The sparse-density analysis is based on the assumption that a freely refined (*xyzB*) atom with no chemical identity approaches a correct atomic position in the structure. Therefore, the method provides excellent results at a crystallographic resolution of 1.5 Å or better where individual atoms can be distinguished in the density maps. At lower resolution, when the free atoms do not necessarily approach the correct atomic positions during refinement, the method is complemented by an exhaustive side-chain conformational search that improves the performance owing to the larger size of the search objects. The method naturally remains sensitive to the accuracy of the built backbone that affects the side-chain conformations.

Here, we present two new methods incorporated into the *ARP*/*wARP* software that specifically address the protein side-chain assignment in crystallographic structure determination at low resolution, especially when only incomplete, fragmented and often main-chain-only models are available. The methods use *a priori* available structural knowledge exploited through statistics-based classification approaches.

## 2. Materials and methods   

### 2.1. Selection of the training set   

Protein structures were retrieved from the Protein Data Bank (PDB; as of 30 September 2014) using the PDB50 clusters (Berman *et al.*, 2000 ▸). The selection criteria included structures obtained using X-ray crystallography, at a resolution better than 2.5 Å, with a crystallographic *R* factor below 0.25, an *R*_free_–*R*_work_ difference of below 0.05, and clashscore and Ramachandran outlier percentiles of higher than 40% in the PDB validation reports (Read *et al.*, 2011 ▸). From this, we randomly selected 1000 structures and their crystallographic models, denoted as ‘conservatively optimized’, and downloaded them from the *PDB-REDO* server together with the corresponding experimental diffraction data (Joosten *et al.*, 2012 ▸). These 1000 structures constituted the training set and, together with their (2*mF*_o_ − *DF*_c_, α_calc_) maps, were used to train the classifier.

### 2.2. Selection of the test set   

Protein structures were taken from the PDB (as of 6 April 2018) using the PDB50 clusters (Berman *et al.*, 2000 ▸). The selection criteria were the same as for the training set (Section 2.1) except that the resolution limit for the X-ray data was broadened to the 2.0–4.0 Å range. The structures present in the training set were excluded and the remaining structures were divided into two test sets: test set I containing 8296 structures within the 2.0–3.0 Å resolution range and test set II containing 752 structures with data below 3.0 Å resolution. To balance the number of structures in the two test sets, test set I was reduced by a random sampling so that 80 structures were selected from each of the ten equal-width resolution bins (within the 2.0–3.0 Å resolution range), resulting in a total of 800 structures in test set I.

The protein structures in both test sets were automatically built by *ARP*/*wARP* v. 8.0 starting from the deposited models. To keep the test sets unbiased to side-chain docking, the sequence information was not used during this model-building step. Following the rationale for the use of the ‘top 50%’ statistics for a web service described in Langer *et al.* (2008 ▸), the top 50% of the built models with the highest model completeness were kept for the analysis presented in this work. There were thus 400 structures in test set I and 375 structures in test set II. The selection of structures from test set I (2.0–3.0 Å range) was performed in equal-width resolution bins as above.

For the evaluation of the developed methods, we used density maps computed from the experimental structure-factor amplitudes and the model-calculated phases with a significant, 40°, uniform random phase error added to all reflections regardless of their structure-factor amplitudes and resolution. For the phases of acentric reflections a random phase error uniformly distributed within the range from −80° to 80° was added. For centric reflections a phase error of 180° was introduced with a probability of 40/180 = 0.22.

### 2.3. Model-quality assessment   

The models built with *ARP*/*wARP* for the test sets were compared with the deposited structures. We define a residue as being built correctly if its C^α^ atom is within a distance of 1.0 Å from the corresponding C^α^ atom in the reference model and if the chain direction of the fragment containing this C^α^ atom is correct. A residue is defined to be ‘correctly docked’ or just ‘docked’ to the sequence if it also has the same side chain built as the corresponding residue in the reference model.

### 2.4. Selecting polypeptide fragments from the training set   

C^α^ atoms from continuous protein chain fragments with a length of between five and nine residues were extracted from the training set and grouped by their mutual structural similarity. For each length the fragments were processed sequentially as follows. The first fragment was assigned as the representative of the first group. The next fragment was added to one of the existing groups if it was structurally similar to its representative. Otherwise, it was assigned as the representative of a new group. Structural similarity between two fragments was evaluated by superimposing them on pairs of four C^α^ atoms (two C^α^ atoms at each fragment termini). Fragments were regarded as structurally similar if, after superposition, the distances between all pairs of corresponding C^α^ atoms were lower than 1.0 Å. For over 700 000 fragments of each length in the training set this grouping procedure yielded 481, 5359, 28 186, 73 904 and 121 107 representative fragments with lengths of five, six, seven, eight and nine C^α^ atoms, respectively. For each group a representative fragment and the size of the group were stored in the database.

### 2.5. Sequence-independent loop building   

**Figure 1:**
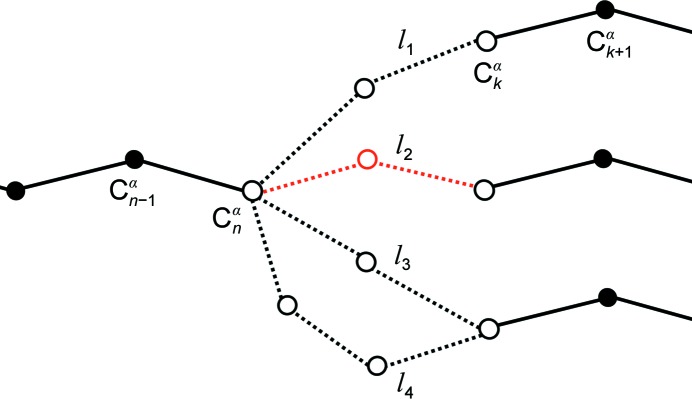
Schematic representation of the sequence-independent loop-building algorithm. Loop candidates {*l*_1_, *l*_2_, *l*_3_, …, *l*_*N*_} are shown with open circles and dashed lines. The most likely loop candidates are built and the loop with the highest *ARP*/*wARP* tracing score above a standard threshold is kept (*l*_2_; marked in red in the figure).

For sequence-independent loop building, we developed an algorithm that identifies plausible connections between the built main-chain fragments before they are docked into the sequence. For each main-chain fragment, two terminal C^α^ atoms are selected. For each terminal C^α^ atom the terminal C^α^ atoms from all other main-chain fragments (including their crystallographic symmetry mates) within a 10 Å radius are considered. Chain directions are ignored at this stage. For each pair of termini a vector of pairwise distances between two terminal C^α^ atoms at each end of the gap (denoted C^α^_*n*−1_, C^α^_*n*_, C^α^_*k*_ and C^α^_*k*+1_ in Fig. 1 ▸) is computed. These distance vectors are then compared with those in the database of representative fragments. The representative fragments of different geometries and lengths are selected as a set of loop candidates {*l*_1_, *l*_2_, …, *l*_*N*_} provided that the length of the difference between the two distance vectors is below 2.0 Å.

The C^α^ atoms in each loop candidate from the set {*l*_1_, *l*_2_, …, *l*_*N*_} are supplemented with additional points: two evenly distributed points are placed between each pair of successive C^α^ atoms. These additional points represent the approximate positions of C and N main-chain atoms. As a result, each loop candidate of *n* residues is represented by a set of 3*n* − 2 points *l*_*i*_ = {*x* *_i_* … *x*_3*n*−2_}.

Each loop candidate with |*l_i_*| points is assigned a statistical score that describes the probability of obtaining its observed match to the density ρ,
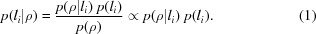



We assume that the probability of the data *p*(ρ) for all candidate loops is the same and therefore can be ignored. To estimate the probability of selecting a given loop candidate by chance, *p*(*l*_*i*_), we use the size of the group to which this fragment belongs normalized by the total number of fragments in the training set. The probability of observing a map density ρ given a loop candidate *p*(ρ|*l*_*i*_) is approximated by the probability of a sum of map densities at its |*l*_*i*_| points being higher than the sum of the same number of map points taken at random locations within a 10 Å radius from the geometric loop centre. As the number of points in the loop is relatively large (at least 13) we approximate the distribution of the sum of the densities at random map points with a Gaussian function, which then simplifies the expression of the combined probability,
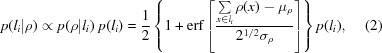
where erf denotes the error function (Glaisher, 1871 ▸) and μ_ρ_ and σ_ρ_ are the mean and standard deviation of a distribution of the sum of |*l_i_*| random values of the density map. Eight top-scored candidate loops are built together with the flanking main-chain fragments using the standard *ARP*/*wARP* main-chain tracing algorithm (Morris *et al.*, 2002 ▸). The loop with the highest score above a default *ARP*/*wARP* threshold is kept.

### 2.6. Side-chain density descriptors   

A side-chain descriptor is required by the developed method to recognize a residue type in the electron density.

As a first step, we took all side-chain conformations for all residue types from the top500 rotamers library (Lovell *et al.*, 2000 ▸) and aligned them by superimposition on their N, C^α^ and C main-chain atoms. We then created a Cartesian grid with a 1.0 Å spacing centred on the C^α^ atom and covering the superimposed side chains. The grid points within 1.0 Å distance from any side-chain atom were selected to form the side-chain grid set. This grid set was superimposed on the N, C^α^ and C main-chain atoms for each residue in the structure being built where the side chain should be. To account for the 1.0 Å grid spacing and to conform to the Shannon sampling theorem, the highest resolution X-ray data for the structure of interest were truncated to 2.0 Å.

**Figure 2:**
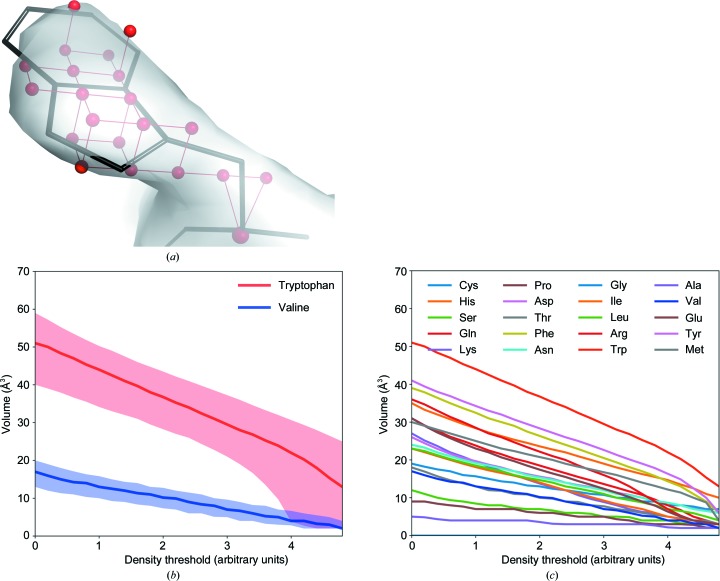
The side-chain descriptor. (*a*) Connected component of a graph (shown in red) built on points sampled in a 2.8 Å resolution 2*mF*_o_ − *DF*_o_ density map at a 1.8σ density level calculated for a refined model of SAM synthetase 2 (PDB entry 2ydx). The side chain of the Trp2016 residue from chain *C* (Trp2016C) is shown in black. (*b*) A comparison between the change in median side-chain density volumes (with 80% confidence intervals) at different map thresholds for tryptophan and valine residues in the training set. (*c*) A comparison of the median side-chain density volumes for all residue types.

An undirected nearest-neighbour graph was constructed from the points within the grid set that fall in electron-density regions above a given density threshold. A breadth-first search of the graph was performed to define connected components of the graph. The number of nodes in the connected component which include the C^α^ atom is taken as an estimate of the side-chain volume at a given density threshold [Fig. 2 ▸(*a*)]. The algorithm starts with the lowest density threshold, which was arbitrarily chosen to be 0.4 r.m.s.d. of the density map. The threshold is then increased with increments of 0.1 r.m.s.d. until this connected component no longer includes the C^α^ atom. This results in a set of side-chain density volumes at different map thresholds.

A number of factors, including the solvent content, the resolution of the X-ray data, the Wilson plot *B* factor and the quality of the phases, may affect the shape of the density distribution (Zwart & Lamzin, 2003 ▸). As a result, the side-chain density volumes estimated at a given threshold may differ from each other for maps calculated from different X-ray data and even for maps at different model-building steps. Therefore, for each residue the set of density volumes, regardless of the number of thresholds that it is built on, is interpolated to yield a vector containing 25 elements that describes the evolution of the side-chain density volume. The vector of 25 elements is referred to as a side-chain descriptor. The descriptors are reasonably distinct for different residue types [Fig. 2 ▸(*b*)] and are used for further classification of the residue type.

The step of interpolating and obtaining a side-chain descriptor is the key part of the method. We have attempted different strategies of selecting the lowest r.m.s.d. density threshold value, including dynamic adjustment based on the estimated solvent content. All of these, after interpolation, resulted in classifiers with a very similar performance. We observed no deterioration of the classifier performance for cases with extreme solvent content.

### 2.7. Residue-type probabilities   

To estimate the probability of a residue type given a side-chain descriptor (a vector with 25 elements described in Section 2.6) we trained a set of support vector machine (SVM) one-versus-all classifiers. A separate classifier was trained to estimate the probability for each of the 20 standard residue types. For a residue in the input main-chain fragment, all 20 classifiers are then used to estimate the probabilities of different residue types, regardless of the protein sequence information. The soft-margin classifiers with a radial basis function kernel were trained using tools from the scikit-learn (Pedregosa *et al.*, 2011 ▸) and LIBSVM (Chang & Lin, 2012 ▸) libraries with a decision function of the form
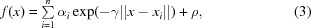
where *x_i_* denotes *n* support vectors defining the separation hyperplane obtained during the training, α_*i*_ are the corresponding weights and ρ is an intercept. To yield the residue-type probability estimates the classifiers were calibrated using a sigmoidal function, which is a common choice for SVM classifiers (Zadrozny & Elkan, 2002 ▸).

Side-chain descriptors were computed for all residues in the training set using the reference models and the corresponding maps calculated with refined-model phases and X-ray data truncated to 2.0 Å resolution. To train the classifier for a given residue type, a random subset of 5000 descriptors for that residue type and a random subset of 5000 descriptors, evenly corresponding to all other residue types, were selected.

### 2.8. Alignment of fragments to the sequence   

For a continuous main-chain fragment in the input model a set of SVM classifiers is used to estimate residue-type probabilities. This yields a statistical scoring matrix, which is used to find alignment probabilities of each fragment to the target sequences. We approximate the probability for the alignment of a fragment as the product of the probability estimates for each residue in the fragment, assuming their independence. Although this assumption is not fully valid, it produces good results at almost no computational cost. We then compare the probability estimate with the distribution of probabilities for an alignment of the same fragment to a random sequence, which we have observed to follow a normal distribution (data not shown). Alignments with a *Z*-score of above 3.7 (corresponding to the 99.99% confidence level) are regarded as reliable and are accepted.

Throughout the model-building process, an evolving main-chain trace may contain mistakes (insertions, deletions or incorrect connections) that may confuse the side-chain assignment step. Therefore, both the complete main-chain fragments from the input model and their continuous subfragments of longer than ten residues are aligned with the target sequence using the residue-type probabilities. For a given fragment, non-overlapping subfragments with the highest probabilities are accepted if their corresponding *Z*-score exceeds a value of 3.7.

### 2.9. Assignment of fragments to subunits   

The assignment of all of the accepted alignments of fragments and their subfragments to the input sequences is carried out using a directed graph analysis. The graph nodes represent aligned fragments and each node may be connected with a directed edge to other nodes if they are assigned to the same chain. The edges correspond to plausible Cartesian distances between the flanking C^α^ atoms of the fragments, given the sequence gap. For example, the graph nodes representing two fragments docked one amino acid apart will be connected with an edge if the Cartesian distance between their termini is within 3.95 Å. This distance limit corresponds to a 99.5% confidence interval of distances between terminal C^α^ atoms in chain fragments from the training set of structures. When graph edges are constructed, plausible paths are enumerated using a depth-first search algorithm. The probability of each path is computed as a product of individual alignment probabilities, and the non-overlapping paths with the highest probabilities are then selected. For model-building cases in which the edges of the graph are short, the method can automatically assign all fragments to their corresponding subunits.

## 3. Results   

### 3.1. Residue-type classifier performance   

**Figure 3:**
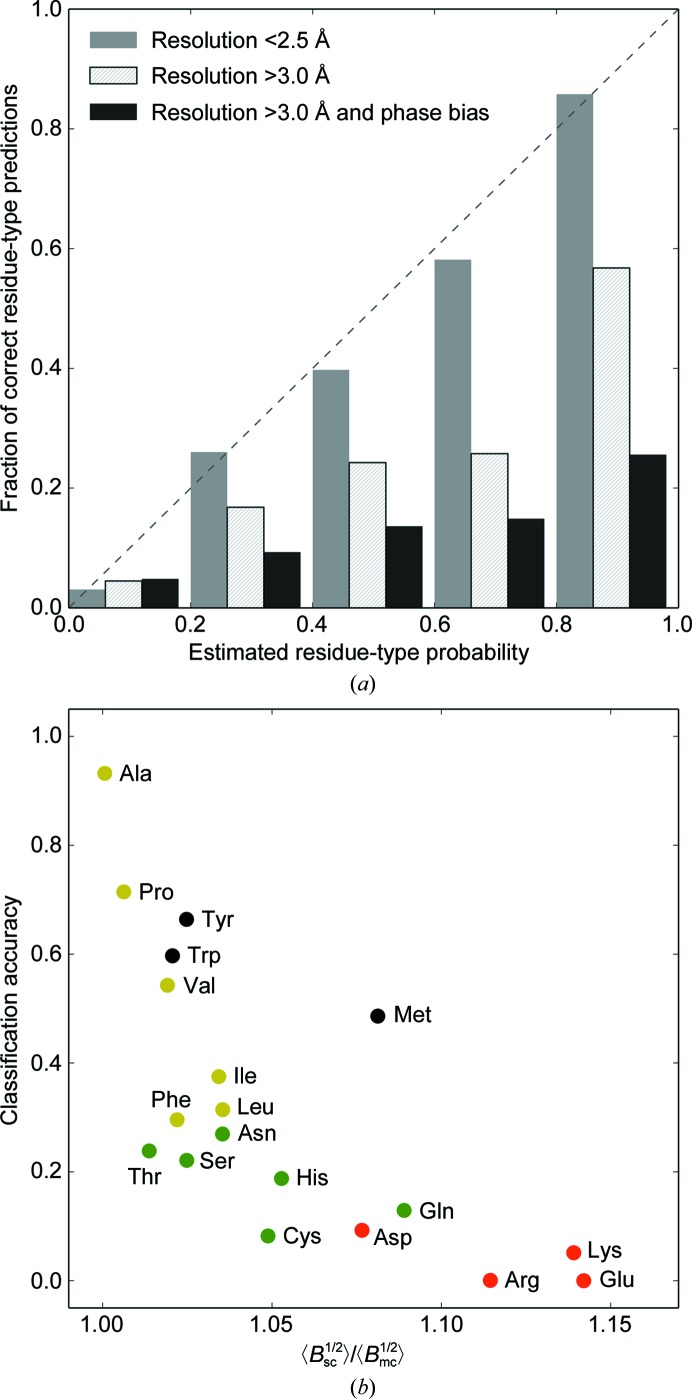
(*a*) Comparison of the estimated residue-type probabilities and the corresponding fraction of correct predictions for different resolution ranges and maps with and without phase bias. (*b*) The accuracy of residue-type classification as a ratio of the side-chain to the main-chain atomic mobility expressed as the ratio of the square roots of their atomic displacement parameters. Residue-type colour codes are as follows: hydrophobic, yellow; charged, red; polar, green; amphiphilic, black.

We studied the performance of the developed method for the estimation of residue-type probabilities using the test-set maps and structures. The results obtained for electron-density maps with a resolution of between 2.0 and 3.0 Å show that the method predicts side-chain probabilities with high accuracy [Fig. 3 ▸(*a*)]. At a lower resolution, and in the presence of additional phase error, the accuracy of the classifier reduces. However, the method is still able to correctly discriminate side-chain types at a resolution as low as 4.0 Å.

We note that the accuracy of the classifier strongly correlates with the side-chain mobility, which we define as the average ratio of the square roots of the side-chain to main-chain atomic displacement parameters [Fig. 3 ▸(*b*)]. We also observe a high accuracy of correct classification for small, buried amino acids that are typically well ordered (*e.g.* 96%, 93% and 72% for glycine, alanine and proline, respectively) as well as for bulky, aromatic residues that are often involved in hydrogen-bond interactions (67% and 60% for tyrosine and tryptophan, respectively). In contrast, the accuracy is lower for solvent-exposed, often disordered residues (*e.g.* 5%, 9% and 18% for lysine, asparagine and histidine, respectively). Methionine may be regarded as an outlier, as despite being poorly ordered compared with other hydrophobic residues [Fig. 3 ▸(*b*)] it is recognized with a high degree of accuracy. This can be attributed to the presence of the S atom in its side chain, which results in a prominent peak in the electron-density map. In contrast, the accuracy of predicting a cysteine (which also contains a S atom) is relatively low and this residue is often misclassified as a valine or a threonine. These three residue types indeed have a similar evolution of their density volume, which is used as the main discriminatory parameter in the method [Fig. 2 ▸(*b*)].

Several side chains look very similar in the electron density owing to the similarity of their chemical structures. Examples include glutamate and glutamine, aspartate and asparagine, and threonine and valine. However, these residues have different mobility properties. For example, glutamate is much more frequently disordered compared with glutamine. Accordingly, glutamine is better recognized in the electron density. Similarly, asparagine is better recognized than aspartate [Fig. 3 ▸(*b*)].

### 3.2. Model-building performance with the new algorithms   

**Figure 4:**
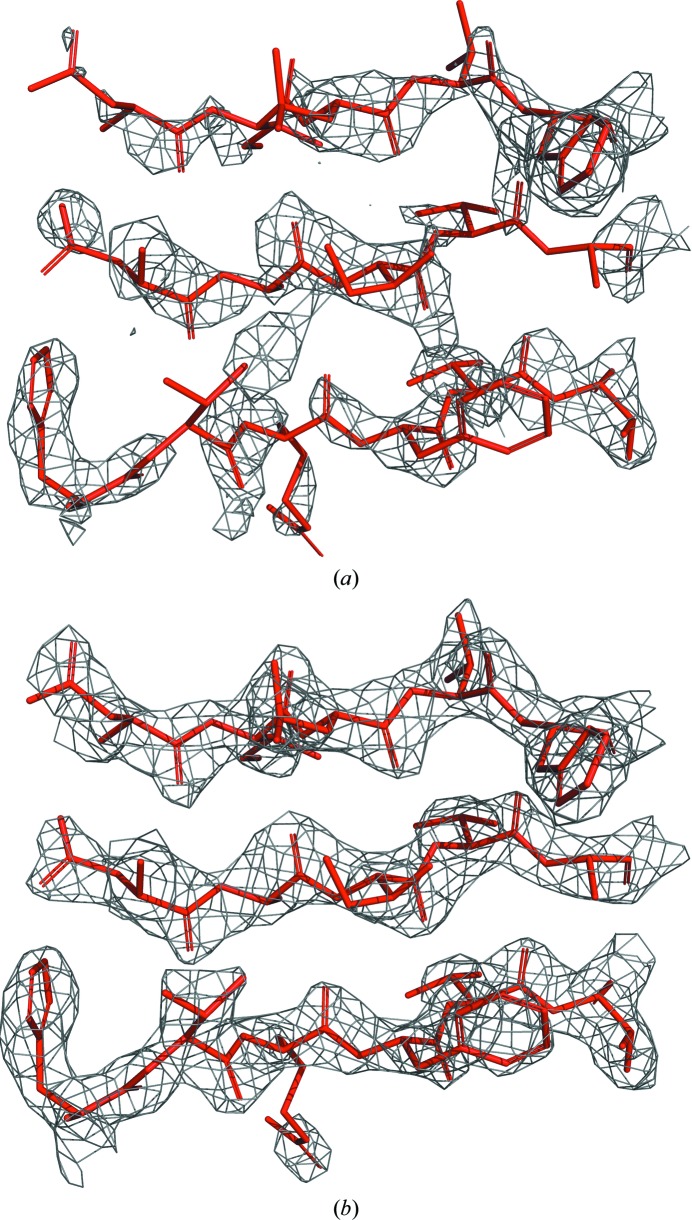
A 2.5 Å resolution density map for the structure of monoxide dehydrogenase (PDB entry 6b6v). (*a*) The map computed from structure factors with a uniform 40° phase error. (*b*) The map with the model phases from the last cycle of model building using *ARP*/*wARP*. The maps are contoured at the 1.5σ density level above the mean. The corresponding model fragments from the deposited structure are shown in red.

To evaluate whether the new methods could provide an improvement in the completeness and quality of automatically built protein models, we incorporated them into the latest version of *ARP*/*wARP* (v. 8.0, released in October 2018) and compared the model-building performances using the default *ARP*/*wARP* parameters. For this, we compared the new (*seqqy*) and the former (*snow*) sequence-assignment methods, and also carried out a comparison using *seqqy* in combination with the new loop-building algorithm (*freeloops*). All model-building tasks were benchmarked on the two test sets with an additional random uniform 40° phase error and the resolution ranges 2.0–3.0 Å and 3.0–4.0 Å (Fig. 4 ▸, see Section 2.2 for details).

**Figure 5:**
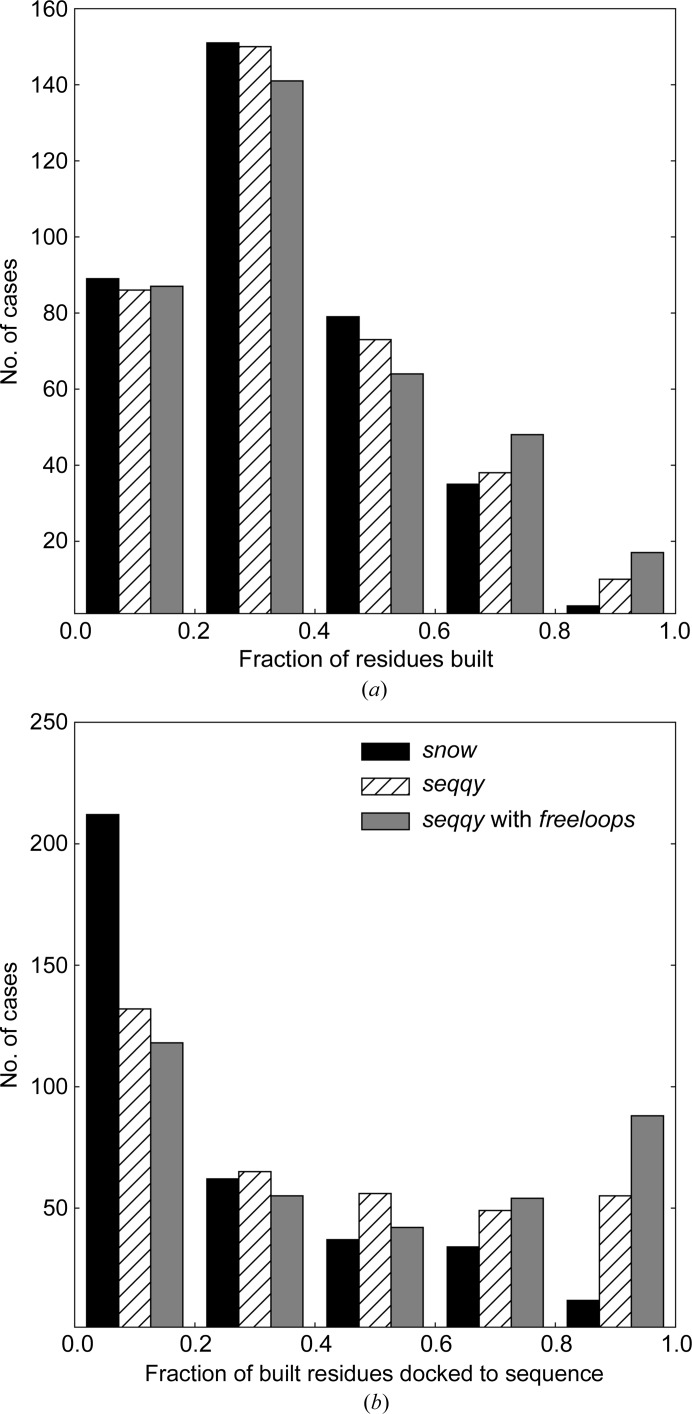
Performance of *ARP*/*wARP* v. 8.0 using the former sequence-docking method (*snow*), the new sequence-docking method (*seqqy*) and a combination of *seqqy* with a sequence-independent loop-building method (*freeloops*) at a resolution lower than 3.0 Å. (*a*) The fraction of residues built. (*b*) The fraction of residues docked into the sequence.

Overall, the use of the new sequence-docking method (*seqqy*) reduces the number of models with a low (below 20%) fraction of side chains built and increases the number of those with high (above 80%) sequence coverage. This is particularly noticeable at a resolutions worse than 3.0 Å, where models may be incomplete and highly fragmented [Fig. 5 ▸(*b*)]. Moreover, the fraction of models with a higher amount of correctly built residues [Figs. 5 ▸(*a*) and 6 ▸(*a*)] is distinctively increased.

### 3.3. Performance of sequence-docking methods   

**Figure 7:**
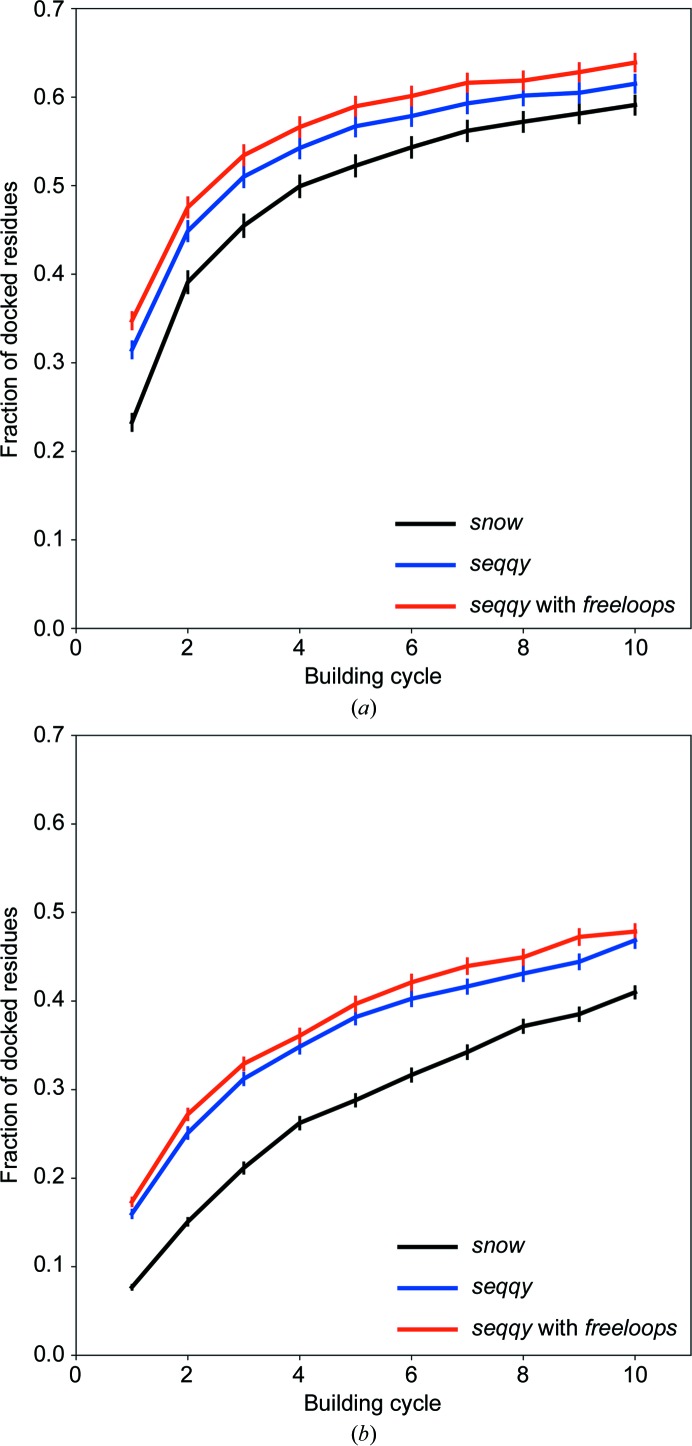
The mean fraction (with the standard deviation of the mean) for residues docked into the sequence as a function of *ARP*/*wARP* building cycle for the test-set cases (*a*) test set I (resolution 2.0–3.0 Å) and (*b*) test set II (resolution worse than 3.0 Å).

To evaluate the performance of the different sequence-assignment methods, we compared the fraction of residues that were correctly docked after each model-building cycle [Fig. 7 ▸(*a*)]. The new sequence-docking algorithm (*seqqy*) outperforms the former one (*snow*) throughout the whole model-building process. This improved performance is pronounced for test set I with X-ray data extending over the range 2.0–3.0 Å. We note that the additional use of the new loop-building algorithm (*freeloops*) further increases the fraction of docked residues. The average chain-fragment length in the 2.0–3.0 Å resolution test set increases from 15 after the first main-chain tracing cycle to 63, 79 and 95 at the end of *ARP*/*wARP* model building when *snow*, *seqqy* and *seqqy* with *freeloops* are used, respectively.

The new sequence-docking method also performs distinctively better for test set II with data in the 3.0–4.0 Å resolution range. A prominent difference in the fraction of correctly docked residues remains throughout all cycles of the model-building process [Fig. 7 ▸(*b*)]. The length of the average chain fragment increases from eight residues after the first main-chain tracing cycle to 12, 17 and 20 in the resulting *ARP*/*wARP* model when *snow*, *seqqy* and *seqqy* with *freeloops* are used, respectively.

### 3.4. Model-building examples from the *ARP*/*wARP* web service   

We tested several examples from a number of cases submitted to the *ARP*/*wARP* web service with a non-restrictive dissemination level. The two that showed the largest improvements are discussed in detail below.

#### 3.4.1. Example 1: NAD-dependent dehydrogenase   

**Figure 8:**
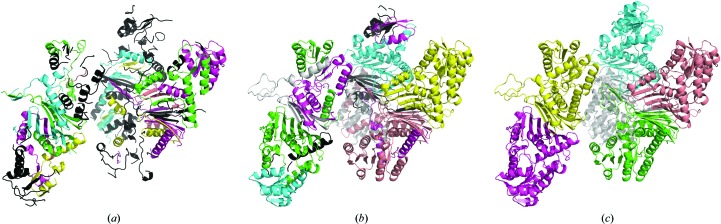
Models of a dehydrogenase at 2.6 Å resolution built with *ARP*/*wARP* v. 8.0 using different sequence-docking methods: (*a*) *snow*, (*b*) *seqqy* and (*c*) *seqqy* in combination with the sequence-independent loop-building method *freeloops*. The parts of the model that it was not possible to dock into the sequence are represented in black, while docked chains are shown in other colours.

The structure was solved using the molecular-replacement pipeline *MrBUMP* (Keegan & Winn, 2007 ▸) and was automatically forwarded to the *ARP*/*wARP* web service for model building. The X-ray data extended to 2.6 Å resolution and contained six molecules of an NAD-dependent dehydrogenase comprising a total of 2046 residues in the asymmetric unit (Fig. 8 ▸). Using the former sequence-docking algorithm (*snow*), *ARP*/*wARP* built 1539 residues distributed in 90 fragments, with 907 residues docked to the sequence. With the new sequence-docking algorithm (*seqqy*) 1958 residues were built in 28 fragments and 1758 residues were docked to the sequence. The use of *seqqy* together with *freeloops* resulted in a more complete model with 2014 residues in nine fragments, with almost all of these, 2009 residues, docked to the sequence. The crystallographic *R*/*R*_free_ factors for the built models (without the free atoms) were 37/45%, 25/31% and 24/29% for the *snow*, *seqqy* and *seqqy* with *freeloops* cases, respectively.

#### 3.4.2. Example 2: AA10 lytic polysaccharide monooxygenase   

**Figure 9:**
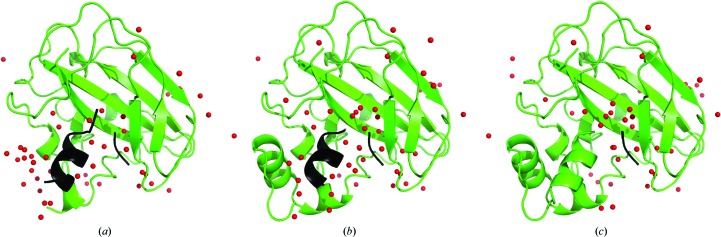
Models of a monooxygenase at 2.2 Å resolution built with *ARP*/*wARP* v. 8.0 using different sequence-docking methods: (*a*) *snow*, (*b*) *seqqy* and (*c*) *seqqy* in combination with the sequence-independent loop-building method *freeloops*. The parts of the model that were not docked into the sequence are shown in black, while docked fragments are shown in green.

This structure contained one molecule of AA10 lytic polysaccharide monooxygenase (183 residues) in the asymmetric unit (Fig. 9 ▸), and the X-ray data extended to 2.2 Å resolution. The structure was solved by MR using *Phaser* (McCoy *et al.*, 2007 ▸) and the model was deposited as PDB entry 6if7 (Yadav *et al.*, 2019 ▸). The authors also attempted structure solution using the MR pipeline *BALBES* (Long *et al.*, 2008 ▸), which solved the structure using *MOLREP* (Vagin & Teplyakov, 2010 ▸) and forwarded it to the *ARP*/*wARP* web service for model building. Using the former sequence-docking algorithm (*snow*), *ARP*/*wARP* built 161 residues in three chain fragments, with 147 residues docked to the sequence. Using the new sequence-docking algorithm (*seqqy*) 176 residues were built, also in three fragments, and 164 residues were docked to the sequence. The use of *seqqy* together with *freeloops* resulted in an even more complete model with 178 residues in two fragments, and almost all of them, 174 residues, were docked to the sequence. The crystallographic *R*/*R*_free_ factors for the built models (without the free atoms) were 31/36%, 27/30% and 25/29% for the *snow*, *seqqy* and *seqqy* with *freeloops* cases, respectively.

## 4. Discussion and conclusions   

In this work, we have presented two novel methods implemented within the automated protein model-building module of *ARP*/*wARP* which provide an increase in the completeness of the automatically built protein models within a wide resolution range.

The new sequence-docking method reported here, *seqqy*, is less sensitive to the accuracy of the model backbone compared with the initial method, *snow*. *seqqy* does not explicitly use the positions of free atoms and thus yields reliable residue-type predictions for maps at lower resolution and in the presence of phase error. The method provides reliable residue-type predictions for individual amino acids. The predictions are particularly accurate for small uncharged residues and side chains containing large rings, which are typically well defined in the density. However, and similar to *snow* and other side-chain docking methods (Cowtan, 2008 ▸), the performance of *seqqy* is reduced for side chains with higher mobility, as these have less defined density support. Examples include the long, often disordered side chains of arginine and lysine residues.

We note that we trained a residue-type classifier on medium-resolution maps (2.5 Å and better) and used it for maps at all resolution ranges as discussed in this paper. This is due to our observation that the density maps at a resolution of 2.5 Å and worse are more noisy in the sense of producing less accurate estimates of side-chain density volumes and thus requiring more support vectors than at higher resolution. An excessive number of support vectors may lead to overfitting and worse classification properties of the SVM classifier (Cortes & Vapnik, 1995 ▸). We also note that although at lower resolution [see, for example, Fig. 3 ▸(*a*)] the residue-type probabilities are overestimated on average, their relative mutual correspondence still allows the side-chain types to be correctly discriminated (data not shown). The development of a more robust classifier trained on lower resolution density maps could be a possible direction of future research.

The performances of *ARP*/*wARP* v. 8.0 using the original sequence-docking method (*snow*), the new method (*seqqy*) and *seqqy* with the new loop-building method *freeloops* were compared using a large set of deposited structures. The starting maps for model building used model-calculated phases that were significantly distorted with intentional random bias. These tests provide a convenient method for a detailed, large-scale analysis of model-building performance at different resolutions of the X-ray data and qualities of the available phases. The demonstrated examples of crystal structures submitted for model building to the *ARP*/*wARP* web service supported the conclusions derived from the benchmarking using the test sets.

The new sequence-assignment and side-chain building method clearly outperforms the original method in interpreting noisy and lower resolution maps. The loop-building method, *freeloops*, further improves the quality of the built models when the overall model completeness is relatively high. We attribute this to the fact that in its current application *freeloops* can only build relatively short loops, which do not really occur in a fragmented structure of low completeness. A fragment library with longer loops could potentially improve the performance at a higher computational cost. Therefore, a natural extension of the method would be to use fragments from a set of structural homologues pre-selected using the target sequence. In principle, this would not only increase the performance of the method (a smaller database) but would also enable the building of longer loops (more accurate fragments). In fact, the number of macromolecular models available in the PDB should make this feasible and applicable for many new crystal structures.

The current implementation of the residue-type classifier provides very encouraging results. It may, however, occasionally misclassify residues with a similar side-chain volume but different topologies (for example cysteine and valine). If the protein sequence is known, a local misassignment should be corrected by docking of the fragment to the sequence. Nevertheless, a potential extension of the presented method­ology would include analysis of the density clusters not only when they include the C^α^ atoms but also when they become disconnected from the main chain. This could help to classify side chains containing sulfur atoms (cysteine and methionine) provided that their density does not overlap with other high-density peaks.

The presented methods for side-chain and loop building in electron-density maps, *seqqy* and *freeloops*, improve the performance of automated model building with *ARP*/*wARP* at medium and low crystallographic resolutions. Therefore, their default use for protein model building with *ARP*/*wARP* may be recommended at resolutions worse than 1.5 Å.

## 5. Implementation and availability   

The methods have been implemented in *ARP*/*wARP* v. 8.0 (http://www.arp-warp.org) with the use of the *CCP*4 (Winn *et al.*, 2011 ▸) and *cctbx* (Grosse-Kunstleve *et al.*, 2002 ▸) utilities and libraries. The benchmarks were performed using the *GNU parallel* software (Tange, 2015 ▸).

## Figures and Tables

**Figure 6:**
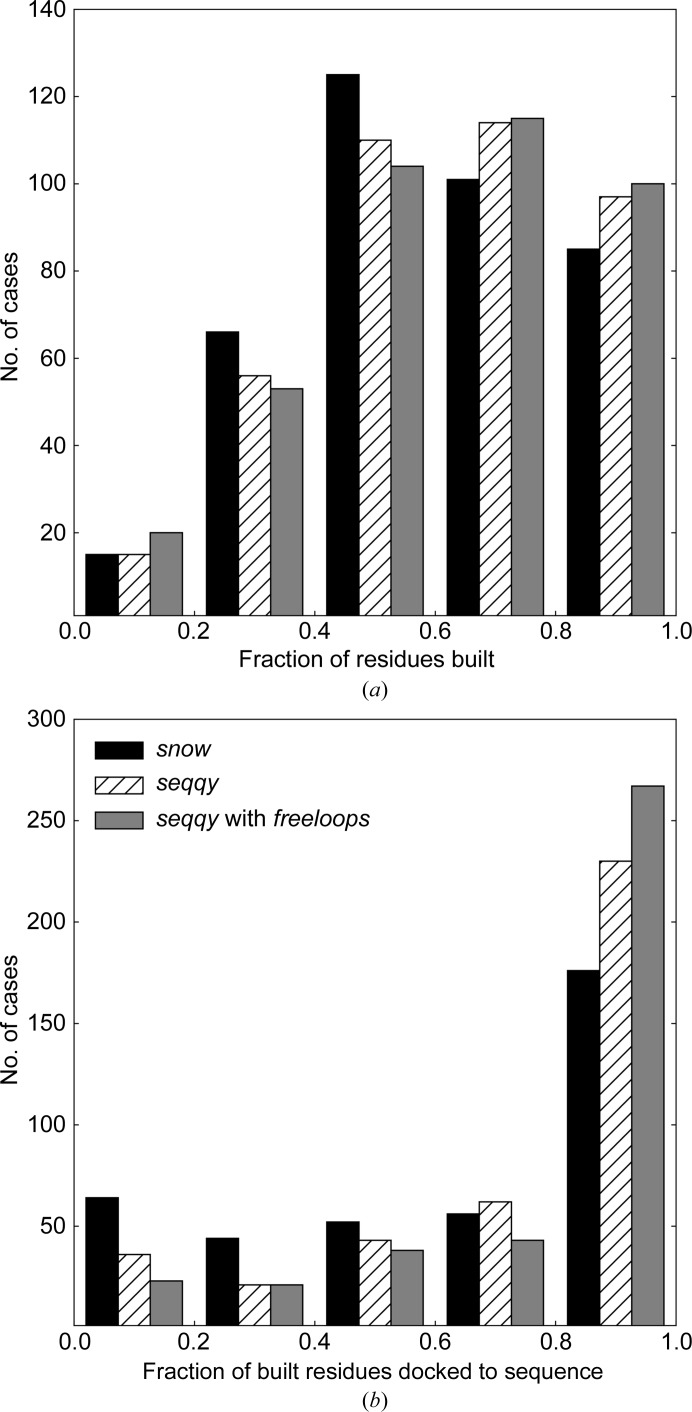
Performance of *ARP*/*wARP* v. 8.0 using the former sequence-docking method (*snow*), the new sequence-docking method (*seqqy*) and a combination of *seqqy* with a sequence-independent loop-building method (*freeloops*) at a resolution between 2.0 and 3.0 Å. (*a*) The fraction of residues built. (*b*) The fraction of residues docked into the sequence.
